# Hypoglycemia in Terminally Ill Patients with Cancer with a History of Diabetes Mellitus Admitted to a General Ward: A Retrospective Observational Study

**DOI:** 10.1089/pmr.2024.0008

**Published:** 2024-08-23

**Authors:** Ryota Yanaizumi, Yusuke Nagamine, Shinsuke Harada, Takahisa Goto

**Affiliations:** ^1^ Department of Anesthesiology, Yokohama City University Medical Center, Yokohama, Japan.; ^2^ Department of Anesthesiology and Critical Care Medicine, Yokohama City University Hospital, Yokohama, Japan.; ^3^ Yu Home Care Clinic Asahi, Yokohama, Japan.

**Keywords:** cancer patient, diabetes mellitus, general ward, hypoglycemia, palliative care

## Abstract

**Background:**

Poor glycemic control may be a risk factor for hypoglycemia in terminally ill patients with cancer with a history of diabetes mellitus (DM). However, no guidelines have been established for achieving glycemic control in this patient population, and epidemiological information remains lacking.

**Objectives:**

We aimed to investigate the prevalence of hypoglycemic episodes and provide epidemiological information on hypoglycemia in terminally ill patients with cancer with a history of DM admitted to a general ward.

**Design:**

This was a single-center, retrospective, observational study.

**Setting/Subjects:**

This study enrolled terminally ill patients with cancer with a history of DM, receiving palliative care at a hospital in Japan between January 2017 and July 2022.

**Measurements:**

Data extracted from the patients’ medical records were age, sex, body mass index, primary cancer, liver metastases, dialysis status, Eastern Cooperative Oncology Group performance status score, type and duration of DM, HbA1c level, and use of diabetes medications (antihyperglycemic agents and types and insulin) at the time of initial visit within 180 days of death.

**Results:**

Among the 104 patients included in the analysis, hypoglycemic episodes occurred in 36 patients (34.6%). The total number of hypoglycemic episodes was 132, and the median number of hypoglycemic episodes for each patient during hospitalization was 2.5 (interquartile range, 1–6).

**Conclusions:**

The prevalence of hypoglycemia in terminally ill patients with cancer with a history of DM who were admitted to a Japanese general ward was 34.6%. Further studies are needed to determine the frequency of hypoglycemia because of overtreatment in this patient population.

## Key Message

The prevalence of hypoglycemia in terminally ill patients with cancer with a history of diabetes mellitus who were admitted to a Japanese general ward was 34.6%. This finding suggests that further studies are warranted to elucidate the risk factors for hypoglycemia in this patient population.

## Introduction

Cancer and diabetes mellitus (DM) are the leading causes of death worldwide, and the number of individuals with both diseases has been increasing.^1^ DM is six times more prevalent in patients with cancer than in other populations.^2^ Therefore, health care providers working with patients with cancer have a high probability of encountering patients with diabetes.^1^

The goal of DM treatment in a normal population is the prevention of long-term micro- and macro-vascular complications, metabolic abnormalities, and symptoms caused by hyperglycemia, in addition to the prevention of sudden death and symptoms caused by hypoglycemia. However, the goal of treatment is not the prevention of long-term vascular complications in patients with poor prognosis, and the treatment goals for hyperglycemia tend to vary.^3^ The primary objective of treatment in such populations should be improving the quality of life (QOL), alleviating cancer-related symptoms, and avoiding unnecessary treatment-related adverse events, especially in patients with advanced cancer who have a median survival period of 6–12 months.^4^ Thus, prolonging life and preventing disability should be secondary goals in such patient populations.^5^ Moreover, unnecessary administration of drugs can also be a financial burden for patients with cancer who have a poor prognosis.^4^

Expert guidelines for DM management in hospice/palliative care recommend setting modest glycemic targets and implementing dietary restrictions and glycemic monitoring.^3,6^ The primary goal of treatment is to avoid the incidence of hypoglycemia, which can result in distress and sudden death.^3,7^ With the rise in the incidence of cancer and diabetes, the management of DM and glycemic control in patients with terminal cancer has become increasingly important in recent years. Patients with end-stage terminal cancer, as well as DM, are managed by different specialties.^8^ Studies involving such patient populations have been scarce.^1,8–12^ Moreover, epidemiological information, such as the prevalence of hypoglycemia and patient background, is not known.

Therefore, this study aimed to investigate the prevalence of hypoglycemia and episodes of hypoglycemia in terminally ill patients with cancer with a history of DM who were admitted to a general ward and gather epidemiological information.

## Methods

### Study design, setting, and patients

This was a single-center, retrospective, observational study of terminally ill cancer patients with a history of DM-seeking treatment from the palliative care team (PCT). Patients with cancer who consulted the PCT at a tertiary care center in Japan between January 2017 and July 2022 were included in this study. Patients with cancer aged ≥18 years who had a history of DM were included. The blood glucose levels were measured during hospitalization at least 180 days prior to death. Patients with unknown outcomes after discharge, patients with a survival duration of ≥181 days from the date of discharge, and patients who did not undergo blood glucose level measurements during hospitalization were excluded from the study.

Patients with “DM” listed in the insurance or medical history in the electronic medical record were defined as patients with preexisting DM. The blood glucose levels were assessed via a bedside fingerstick glucose monitoring method. The participating Medical Center is an academic general hospital with a tertiary emergency center and has no wards or beds specifically assigned to palliative care patients. All patients with terminal cancer were assigned to a primary physician/surgeon or an appropriate specialty cancer team. Palliative care was administered at the discretion of the primary doctor/team or upon patient request. In Japan, the primary reason for consulting PCTs is for symptom control, especially pain management.

### Patient characteristics

Data regarding the following factors were extracted from the electronic medical records of the patients: age, sex, body mass index (BMI), primary cancer, liver metastases, dialysis status, Eastern Cooperative Oncology Group performance status (ECOG-PS) score, type and duration of DM, HbA1c level, and the use of diabetes medications (antihyperglycemic agents and their types and insulin) at the time of first admission within 180 days of death.

### Outcome

The primary outcome of this study was to assess the prevalence of hypoglycemia. Hypoglycemia was defined as a blood glucose level <70 mg/dL or within the range of 70–80 mg/dL with evident hypoglycemic symptoms.^13^ In contrast, the International Hypoglycemia Study Group recommends classifying hypoglycemia in clinical trials as a blood glucose level <54 mg/dL, which is sufficiently low to indicate clinically significant hypoglycemia.^14^ Therefore, we also defined clinically significant hypoglycemia as a blood glucose level <54 mg/dL. The secondary outcome was to determine whether patients who experienced hypoglycemia also had episodes of diabetic ketoacidosis or hyperglycemic hyperosmotic syndrome, which are serious complications associated with hyperglycemia (blood glucose levels of >300 mg/dL at any given time).^15^ The diagnosis of diabetic ketoacidosis or hyperglycemic hyperosmotic syndrome was based on the Japanese Clinical Practice Guideline for Diabetes.^13^ In addition, as a sensitivity analysis, the prevalence of hypoglycemia was recalculated under the hypothetical assumption that those terminally ill patients with cancer who were excluded because they did not undergo blood glucose measurement on admission did not have hypoglycemia.

### Statistical analysis

All statistical analyses were performed using JMP version 16 (SAS Institute Inc., Cary, NC, USA). Categorical and numerical data of the hypoglycemic and nonhypoglycemic groups were analyzed using the chi-square and Mann–Whitney *U* tests, respectively. Statistical significance was set at *p* < 0.05. Sample size calculation was not performed as this was a descriptive epidemiological study. The prevalence proportion of hypoglycemia was calculated by dividing the number of patients with hypoglycemic episodes by the total number of patients. The incidence rate of hypoglycemia was calculated by dividing the total number of hypoglycemic episodes by the sum of observation periods (days) for each patient, then expressed as per 100 person-days.

### Ethical considerations

This retrospective observational study was approved and the requirement for obtaining written informed consent was waived by the Ethics Committee. The patients were given the opportunity to withdraw consent on the website through an opt-out option. This study was conducted in accordance with the Declaration of Helsinki and Good Clinical Practice Guidelines. This observational study complied with the Strengthening the Reporting of Observational Studies in Epidemiology guidelines.^16^

## Results

**FIG. 1.**
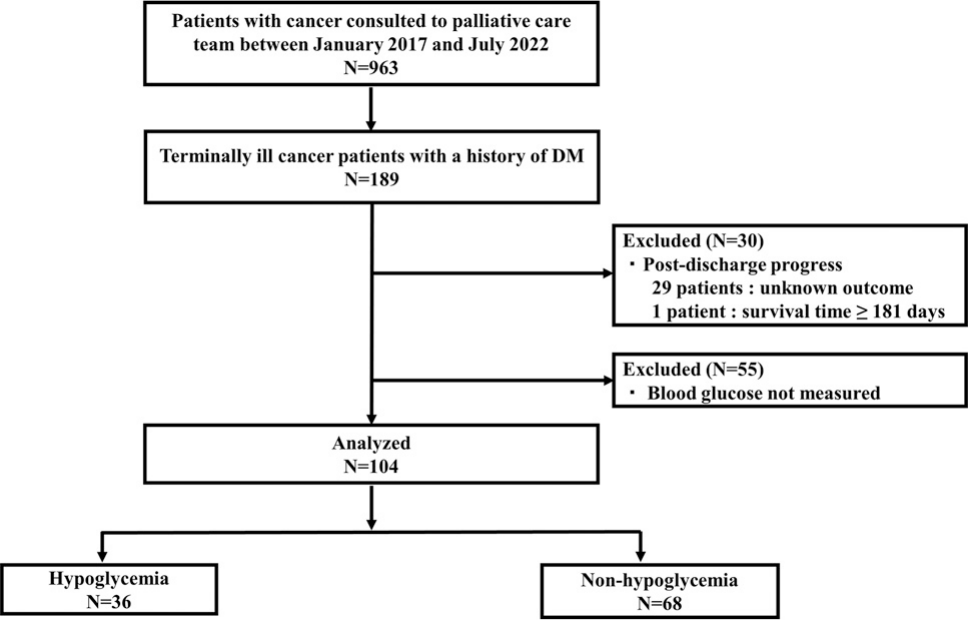
Flow chart of the study. DM, diabetes mellitus.

Among the 963 patients with cancer who consulted the PCT after admission, 189 patients had a history of DM. Twenty-nine patients had an unknown outcome after discharge. One patient had a survived duration of ≥181 days after discharge. Fifty-five patients did not undergo blood glucose level measurements during hospitalization for 180 days before death. Thus, 104 terminally ill patients with cancer were included in the study. Among these 104 patients, 36 had hypoglycemia, resulting in a prevalence proportion of hypoglycemia of 34.6% (Fig. 1). Furthermore, our study excluded 55 patients who did not undergo blood glucose measurement on admission. If we hypothetically assume that these terminally ill cancer patients did not have hypoglycemia, the prevalence of hypoglycemia would be 22.6% [36 patients with hypoglycemia/(104 patients + 55 patients)] in our sensitivity analysis. In addition, 18 patients had clinically significant hypoglycemia (blood glucose <54 mg/dl), resulting in a prevalence of clinically significant hypoglycemia of 17.3%.

The baseline characteristics of the 104 patients (data of the first hospitalization within 180 days prior to the date of death) are tabulated (Table 1). The median age of the participants was 71.0 years (interquartile range [IQR], 66.3–77.0 years) and 84 patients (80.8%) were men. The median BMI was 21.7 kg/m^2^ (IQR, 19.2–24.1 kg/m^2^). The primary tumor sites were the lung (23.1%), pancreas (17.5%), stomach (9.6%), colon and rectum (8.7%), and liver (2.9%). Forty-six patients (44.2%) had liver metastasis, and four patients (3.8%) underwent dialysis. The ECOG-PS scores were 3 and 2 in 37 (35.6%) and 32 (30.8%) patients, respectively. All patients had type 2 DM (100.0%). The median duration of DM was 96 months (IQR, 19–168 months). The median HbA1c level was 6.9% (IQR, 6.4–7.6%). Fifty-four patients (51.9%) were receiving antihyperglycemic agents (sulfonylurea, 4.8%; glinide, 9.6%; dipeptidyl peptidase (DPP)−4 inhibitor, 39.4%; biguanide, 11.5%; sodium-glucose transport protein (SGLT)-2 inhibitor, 5.8%; glucagon-like peptide (GLP)-1 receptor agonist, 3.8%). Seventy-four patients (71.2%) were receiving insulin.

**Table 1.** Baseline Characteristics of the 104 Patients Included in This Study

	Total (*n* = 104)
	Number
Age, years	
Median (IQR)	71.0 (66.3–77.0)
Sex	
Men (%)	84 (80.8)
BMI, kg/m^2^	
Median (IQR)	21.7 (19.2–24.1)
Primary tumor site	
Lung, number (%)	24 (23.1)
Pancreas, number (%)	18 (17.3)
Stomach, number (%)	10 (9.6)
Colon-rectum, number (%)	9 (8.7)
Liver, number (%)	3 (2.9)
Others, number (%)	40 (38.5)
Liver metastasis, number (%)	46 (44.2)
Patients with dialysis (%)	4 (3.8)
ECOG Performance Status Scale	
1 (%)	31 (29.8)
2 (%)	32 (30.8)
3 (%)	37 (35.6)
4 (%)	4 (3.8)
Patients with type 1 DM (%)	0 (0)
Patients with type 2 DM (%)	104 (100)
Duration of DM, months	
Median (IQR)	96 (19–168)
HbA1c, %	
Median (IQR)	6.9 (6.4–7.6)
Diabetes medications	
Antihyperglycemic agent^a^ (%)	54 (51.9)
Sulfonylurea (%)	5 (4.8)
Glinide (%)	10 (9.6)
DPP-4 inhibitor (%)	41 (39.4)
Biguanide (%)	12 (11.5)
SGLT-2 inhibitor (%)	6 (5.8)
GLP-1 receptor agonist (%)	4 (3.8)
Insulin (%)	74 (71.2)
None (%)	0 (0)

^a^ Duplicates were present.

BMI, body mass index; DM, diabetes mellitus; DPP, dipeptidyl peptidase; ECOG, Eastern Cooperative Oncology Group; GLP, glucagon-like peptide; IQR, interquartile range; SGLT, sodium-glucose transport protein.

Details regarding each hypoglycemic episode in the 36 patients with hypoglycemia are tabulated (Table 2). The total number of hypoglycemic episodes was 132. The incidence rate of hypoglycemia was 1.3 per 100 person-days. The median number of hypoglycemic episodes during the period of hospitalization was 2.5 (IQR 1–6). The median minimum blood glucose level was 58 mg/dL (IQR 50–66 mg/dL) for all 132 hypoglycemic episodes and 54 mg/dL (IQR 50–65.5) for the 36 patients. The ECOG-PS scores at the time of each hypoglycemic episode were 4, 3, and 2 in 78 (59.1%), 48 (36.4%), and six (4.5%) patients, respectively. The nutrient pathways at the time of each hypoglycemic episode were as follows: enteral (oral intake [34.8%] and tube feeding [6.8%]), parenteral, peripheral venous infusion (62.1%), and central venous hyperalimentation (12.1%) [duplicates present]. The patients were receiving antihyperglycemic agents (sulfonylurea, 0%; glinide, 0.8%; DPP-4 inhibitor, 13.6%; biguanide, 0%; SGLT-2 inhibitor, 0%, GLP-1 receptor agonist, 8.3%) at the time of 29 episodes (21.9%). A total of 104 (78.8%) episodes involved patients who were receiving insulin. Twenty-eight episodes (21.2%) were reported in patients who were not receiving anti-hyperglycemic agents or insulin. The median number of days from the first hypoglycemic episode during hospitalization to death was 28.5 (IQR 10.0–75.0 days). Twelve of the 36 patients with hypoglycemia had experienced at least one episode of hyperglycemia (blood glucose levels of >300 mg/dL at any given time). None of the patients were diagnosed with diabetic ketoacidosis or hyperglycemic hyperosmotic syndrome, which are serious complications of hyperglycemia. In addition, details regarding each hypoglycemic episode in the 18 patients with clinically significant hypoglycemia (blood glucose <54 mg/dL) are provided in Supplementary Table S1.

**Table 2.** Details of Hypoglycemic Episodes in the 36 Patients with Hypoglycemia

	Number
Total number of hypoglycemic episodes, episode	132
Incidence rate of hypoglycemia, per 100 person-days	1.3
Hypoglycemic episodes per patient, episode	
Median (IQR)	2.5 (1–6)
Median blood glucose levels during all 132 episodes of hypoglycemia, mg/dL	
Median (IQR)	58 (50–66)
Median lowest blood glucose level in each of the 36 patients, mg/dL	
Median (IQR)	54 (50–65.5)
ECOG Performance Status Scale (each ECOG performance status at the occurrence of hypoglycemia)	
1 (%)	0 (0)
2 (%)	6 (4.5)
3 (%)	48 (36.4)
4 (%)	78 (59.1)
Nutrient pathways^a^ (each nutritional pathway at the occurrence of hypoglycemia)	
Enteral nutrition	
Oral intake (%)	46 (34.8)
Tube feeding (%)	9 (6.8)
Parenteral nutrition	
Peripheral venous infusion (%)	82 (62.1)
Central venous hyperalimentation (%)	16 (12.1)
Diabetes medications (each DM treatment for the occurrence of hypoglycemia), episode (%)	
Anti-hyperglycemic agent^a^	29 (21.9)
Sulfonylurea (%)	0 (0)
Glinide (%)	1 (0.8)
DPP-4 inhibitor (%)	18 (13.6)
Biguanide (%)	0 (0)
SGLT-2 inhibitor (%)	0 (0)
GLP-1 receptor agonist (%)	11 (8.3)
Insulin (%)	104 (78.8)
None (%)	28 (21.2)
Days from first hypoglycemic episode to death, days	
Median (IQR)	28.5 (10.0–75.0)

^a^ Duplicates were present.

DM, diabetes mellitus; DPP, dipeptidyl peptidase; ECOG, Eastern Cooperative Oncology Group; GLP, glucagon-like peptide; IQR, interquartile range; SGLT, sodium-glucose transport protein.

A comparison between hypoglycemia patients and nonhypoglycemia patients at the time of first admission within 180 days of death is tabulated (Table 3). The percentage of patients receiving DPP-4 inhibitors was significantly lower among patients with hypoglycemia compared with those without hypoglycemia (25.0 vs. 47.1%; *p* = 0.026). In contrast, the percentages of patients receiving sulfonylurea and insulin were higher among patients with hypoglycemia compared with those without hypoglycemia (sulfonylurea 11.1 vs. 1.5%; *p* = 0.033, insulin 91.7 vs. 60.3%; *p* = 0.0003).

**Table 3.** Comparison Between Hypoglycemia Patients and Non-Hypoglycemia Patients at the Time of First Admission Within 180 Days of Death

	Hypoglycemia patients (*n* = 36)	Non-hypoglycemia patients (*n* = 68)	*p* value
	Number	Number	
Age, years			
Median (IQR)	71.0 (65.3–76.5)	70.5 (67.3–77.0)	0.771
Sex			
Men (%)	27 (75.0)	57 (83.8)	0.284
BMI, kg/m^2^			
Median (IQR)	21.0 (18.9–25.2)	22.1 (19.5–24.0)	0.53
Primary tumor site			
Lung, number (%)	12 (33.3)	12 (17.6)	0.076
Pancreas, number (%)	8 (22.2)	10 (14.7)	0.359
Stomach, number (%)	2 (5.6)	8 (11.8)	0.287
Colon-rectum, number (%)	1 (2.8)	8 (11.8)	0.092
Liver, number (%)	0 (0)	3 (4.4)	0.107
Others, number (%)	13 (36.1)	27 (39.7)	0.719
Liver metastasis, number (%)	15 (41.7)	31 (45.6)	0.701
Patients with dialysis (%)	3 (8.3)	1 (1.5)	0.092
ECOG Performance Status Scale			
1 (%)	11 (30.6)	20 (29.4)	0.904
2 (%)	8 (22.2)	24 (35.3)	0.163
3 (%)	16 (44.4)	21 (30.9)	0.172
4 (%)	1 (2.8)	3 (4.4)	0.672
Patients with type 1 DM (%)	0 (0)	0 (0)	
Patients with type 2 DM (%)	36 (100)	68 (100)	
Duration of DM, months			
Median (IQR)	59 (11.3–162)	116 (45.3–168)	0.153
HbA1c, %			
Median (IQR)	7.2 (6.4–7.8)	6.8 (6.4–7.5)	0.269
Diabetes medications			
Antihyperglycemic agent^a^ (%)	15 (41.7)	39 (57.4)	0.127
Sulfonylurea (%)	4 (11.1)	1 (1.5)	0.033
Glinide (%)	4 (11.1)	6 (8.8)	0.709
DPP-4 inhibitor (%)	9 (25.0)	32 (47.1)	***0.026***
Biguanide (%)	4 (11.1)	8 (11.8)	0.921
SGLT-2 inhibitor (%)	1 (2.8)	5 (7.4)	0.314
GLP-1 receptor agonist (%)	3 (8.3)	1 (1.5)	0.092
Insulin (%)	33 (91.7)	41 (60.3)	0.0003
None (%)	0 (0)	0 (0)	

^a^ Duplicates were present.

BMI, body mass index; DM, diabetes mellitus; DPP, dipeptidyl peptidase; ECOG, Eastern Cooperative Oncology Group; GLP, glucagon-like peptide; IQR, interquartile range, SGLT, sodium-glucose transport protein.

## Discussion

Among the 104 terminally ill patients with cancer with a history of DM admitted to a general ward to receive palliative care who were included in this single-center, retrospective, observational study, hypoglycemic episodes occurred in 36 patients, and the prevalence of hypoglycemia was 34.6%. The total number of hypoglycemic episodes was 132, with a median of 2.5 episodes per patient. To the best of our knowledge, this is the first study to report on the incidence of hypoglycemia in terminally ill cancer patients with a history of DM admitted to a general ward.

The objective of treatment for patients with advanced cancer who have a median survival duration of 6–12 months should be maintaining the QOL and relief of cancer-related symptoms, such as pain, as well as the prevention of the incidence of adverse events due to unnecessary treatment.^4^ Expert guidelines on diabetes management in hospice/palliative care recommend gradual glycemic control at the end of life, especially in patients with type 2 DM.^3,6^ The primary goal should be the prevention of hypoglycemia, which can lead to distress and sudden death.^3,7^ In this study, the median number of days from the first hypoglycemic episode during hospitalization to death was 28.5 days. Among the 132 hypoglycemic episodes, patients were receiving insulin at the time of 78.8% of episodes, suggesting that insulin may have been administered to an otherwise unnecessary patient population. It is also important to avoid the adverse effects of hyperglycemia in patients receiving end-of-life care.^10^ In this study, 12 of the 36 patients who experienced hypoglycemic episodes had hyperglycemia (blood glucose >300 mg/dL at any time) at least once; however, none of the patients were diagnosed with diabetic ketoacidosis/hyperglycemic hyperosmotic syndrome, and no adverse effects related to hyperglycemia were observed.

The prevalence of hypoglycemia among terminally ill patients with cancer with a history of DM who were admitted to a general ward was 34.6% in this study. The prevalence of hypoglycemia in patients with diabetes varies widely owing to significant heterogeneity in the definition, measurement, and reporting of hypoglycemia in different studies.^17^ A study conducted in the Netherlands reported that in a group of insulin users with type 2 DM with an average age of 67.2 years receiving usual care, severe hypoglycemia requiring third-party help occurred in 4% of patients per year.^18^ In contrast, a report from Denmark reported that the prevalence of hypoglycemia was 16.5% per year in a group of patients with type 2 DM receiving insulin with an average age in their 60 seconds.^19^ In addition, a retrospective cohort study of patients older than 65 years with type 2 DM admitted to Veterans Affairs nursing homes in the United States showed that the cumulative incidence of hypoglycemia (glucose <70 mg/dL) among all patients, accounting for the competing risk of death, was 12% at 180 days, and that of severe hypoglycemia (glucose <50 mg/dL) was 5%.^12^ Thus, the reported prevalence of hypoglycemia varies between studies. Direct comparisons are difficult to perform as no studies similar to the present study have been conducted on the prevalence of hypoglycemia in terminally ill patients with cancer who were admitted to general wards.

The analysis of all 132 episodes of hypoglycemia in this study revealed that the ECOG-PS score worsened compared with that at the time of initial admission and that more than 60% of the patients were unable to take food orally. Decreased physical activity and the inability to take food orally are recognized risk factors for hypoglycemia in patients with type 2 DM^17^; aligning with the findings of this study. In contrast, the participants had not received insulin or antihyperglycemic agents on the day of the episode in 28 of the 132 hypoglycemic episodes (data not shown). These 28 cases of hypoglycemia were reported in 7 patients, all occurring within the first month of prognosis. Starvation owing to the lack of oral intake and the prolonged effect of the discontinuation of antihyperglycemic agents or long-term insulin preparations may have contributed to the incidence of hypoglycemic episodes. Furthermore, nondiabetic hypoglycemic episodes may have also occurred due to decreased oral intake and impaired gluconeogenesis owing to liver and kidney dysfunction during the terminal stage in patients with cancer.^20^ It is challenging to determine retrospectively whether these episodes were due to drug residuals or nondiabetic causes. In the electronic medical records, only 6 out of the 28 cases of hypoglycemia in these 7 patients had been documented by the diabetologist as likely resulting from long-term residual high-dose sulfonylurea drug use. Caution must be exercised when interpreting these findings, as this study did not compare the circumstances during hypoglycemic episodes with those when hypoglycemia did not occur in the same patient.

The strength of this study lies in the uniqueness of its clinical question. To the best of our knowledge, no study has reported the incidence of hypoglycemia in terminally ill patients with cancer with a history of DM who were admitted to a general ward. Given the increase in the number of patients with cancer as well as diabetes,^1^ the prevention of adverse events induced by hypoglycemia in such populations should be the focus of future studies.

Our study has several limitations. First, this was a single-center retrospective study with a relatively small sample size. Second, 80% of the patients were males, indicating a sex bias. Moreover, the population was also racially biased as only individuals of Japanese ethnicity were included in this study. Third, the frequency of blood glucose measurements may not have been consistent across all patients, leading to potential challenges in accurately assessing blood glucose levels at the onset of hypoglycemia. Last, it is difficult to compare the same patients with and without hypoglycemia because of the retrospective nature of the study. Further, more sophisticatedly constructed studies are needed. For example, an observational study of the prevalence of hypoglycemia and hyperglycemia in terminally ill patients with cancer with a history of diabetes who were using insulin or antihyperglycemic agents and whose blood glucose was controlled only by a sliding scale after hospitalization is warranted.

In conclusion, among 104 terminally ill patients with cancer with a history of DM who were admitted to a general ward for palliative care, hypoglycemic episodes occurred in 36 patients. The prevalence of hypoglycemic episodes was 34.6%. The total number of hypoglycemic episodes was 132, with a median of 2.5 episodes per patient. Further studies are needed to determine the frequency of hypoglycemia because of overtreatment in terminally ill patients with cancer with a history of DM.

## Data Availability

The data supporting the findings of this study are available from the corresponding author upon reasonable request.

## Abbreviations Used

BMI: body mass index
DM: diabetes mellitus
DPP: dipeptidyl peptidase
ECOG-PS: Eastern Cooperative Oncology Group performance status
FS: fingerstick
GLP: glucagon-like peptide
IQR: interquartile range
PCT: palliative care team
QOL: quality of life
SGLT: sodium-glucose transport protein
STROBE: the Strengthening the Reporting of Observational Studies in Epidemiology
