# Supplementary material for: Hypoglycemia in Terminally Ill Patients with Cancer with a History of Diabetes Mellitus Admitted to a General Ward: A Retrospective Observational Study
Source: Palliat Med Rep. 2024 Aug 23;5(1):373–80. doi: 10.1089/pmr.2024.0008 (PMC11392688; doi:10.1089/pmr.2024.0008)
Supplement: Supplementary Table S1 [file pmr.2024.0008_nagamine_supplementary_tables.pdf]

Supplementary Table 1. Details of hypoglycemic episodes in the 18 patients with clinically significant hypoglycemia (blood glucose <54 mg/dl)

	Number
Total number of hypoglycemic episodes, episode	49
Incidence rate of hypoglycemia, 100 person-days	0.5
Hypoglycemic episodes per patient, episode	
Median (IQR)	2 (1–4)
Median blood glucose levels during all 49 episodes of hypoglycemia, mg/dl	
Median (IQR)	48 (38–51)
Median lowest blood glucose level in each of the 18 patients, mg/dl	
Median (IQR)	50 (36.3–52)
ECOG Performance Status Scale (each ECOG Performance Status at the occurrence of hypoglycemia)	
1 (%)	0 (0)
2 (%)	0 (0)
3 (%)	16 (32.7)
4 (%)	33 (67.3)
Nutrient pathways* (Each nutritional pathway at the occurrence of hypoglycemia)	
Enteral nutrition	
Oral intake (%)	13 (26.5)
Tube feeding (%)	3 (6.1)
Parenteral nutrition	
Peripheral venous infusion (%)	29 (59.2)
Central venous hyperalimentation (%)	10 (20.4)
Diabetes medications (each DM treatment for the occurrence of hypoglycemia), episode (%)	
Anti-hyperglycemic agent*	6 (12.2)
Sulfonylurea (%)	0 (0)
Glinide (%)	0 (0)
DPP-4 inhibitor (%)	5 (10.2)
Biguanide (%)	0 (0)
SGLT-2 inhibitor (%)	0 (0)
GLP-1 receptor agonist (%)	1 (2.0)
Insulin (%)	36 (73.5)
None (%)	13 (26.5)
Days from first hypoglycemic episode to death, days	
Median (IQR)	13.5 (4–25)

Abbreviations: IQR, interquartile range; ECOG, Eastern Cooperative Oncology Group; DM, diabetes mellitus; DPP, dipeptidyl peptidase; SGLT, sodium-glucose transport protein; GLP, glucagon-like peptide.

*Duplicates were present
